# Scorpion species of medical importance in the Brazilian Amazon: a review to identify knowledge gaps

**DOI:** 10.1590/1678-9199-JVATITD-2021-0012

**Published:** 2021-09-20

**Authors:** Jonas Gama Martins, Gabrielle Cristina Santos, Rudi Emerson de Lima Procópio, Eliane Candiani Arantes, Karla de Castro Figueiredo Bordon

**Affiliations:** 1 Graduate Program in Genetics, Conservation and Evolutionary Biology (PPG GCBEv), National Institute for Amazon Research (INPA), Manaus, AM, Brazil.; 2 Department of BioMolecular Sciences, School of Pharmaceutical Sciences of Ribeirão Preto, University of São Paulo (USP), Ribeirão Preto, SP, Brazil.; 3 Graduate Program in Biotechnology and Natural Resources of Amazon, University of the State of Amazonas (UEA), Manaus, AM, Brazil.

**Keywords:** Brazilian Amazon, Endemic scorpions, Arboreal scorpions, Venom, Tityus metuendeus, Tityus silvestris, Brotheas amazonicus, Tityus strandi, Tityus apiacas, Tityus obscurus

## Abstract

Scorpionism is a relevant medical condition in Brazil. It is responsible for most accidents involving venomous animals in the country, which leads to severe symptoms that can evolve to death. In recent years, an increase of almost 50% in the incidence of scorpionism has been observed in the Northern Region, where the highest severity of envenoming has been notified since the beginning of the 21^st^ century. This review aims to provide an in-depth assessment of public data and reports on symptoms and epidemiology of envenoming, ecological aspects of scorpions, and characterization of venoms and toxins to access the gaps that need to be filled in the knowledge of the scorpion species of medical importance from the Brazilian Amazon. A systematic search using the string words “Amazon” and “scorpion” was performed on 11 databases. No restriction on date, language or status of the publication was applied. Reports not related to the Brazilian Amazon were excluded. Therefore, 88 studies remained. It is shown that populations of scorpions of medical importance, even of the same species, may present significant toxic variations peculiar to some regions in the Brazilian Amazon, and commercial scorpion antivenoms were not able to shorten the intensity and duration of neurological manifestations in patients stung by *T. silvestris, T. apiacas* or *T. obscurus*. It is also highlighted that the toxins responsible for triggering these alterations have not been elucidated yet and this is a fruitful field for the development of more efficient antivenoms. Furthermore, the geographic distribution of scorpions of the genus *Tityus* in the Brazilian Amazon was revised and updated. The cumulative and detailed information provided in this review may help physicians and scientists interested in scorpionism in the Brazilian Amazon.

## Background

To date, there are 2,584 scorpion species worldwide distributed into 23 families (according to [1] and updated by [2-6]). So far, four scorpion families (Bothriuridae, Buthidae, Chactidae and Hormuridae), 23 genera and about 160 species have been reported in Brazil [7-9], which represents 6.3% of the worldwide diversity of these arachnids.

Scorpionism remains a serious public health problem. More than 1.2 million scorpion stings and 3,000 deaths caused by scorpion envenoming are registered annually worldwide, and about 2.3 billion people live in areas of scorpionism risk [10, 11]. 

Buthidae, the largest of the scorpion families, is distributed in several regions around the world, except Antarctica and New Zealand [3]. This family comprises 1,225 species (updated on January 22^nd^, 2021) [12], including about 50 species considered dangerous to humans [13]. 

Around 95% of the scorpion envenomings are caused by species of the family Buthidae C. L. Koch, which includes the genera *Tityus*, *Centruroides*, *Mesobuthus*, *Parabuthus*, *Leiurus*, *Buthus*, *Hottentota* and *Androctonus* [14]. Scorpions belonging to Chactidae family are capable of causing mild and local toxicity in humans [15, 16]. However, a greater number of scorpion species can be potentially harmful to humans. Thus far, 104 scorpion species have been considered medically significant in the literature. However, there is a lack of reports of symptoms induced by the venom of most of these species [17]. 

Although scorpions are present on all continents, except Antarctica, the severity and incidence of envenoming are higher in the northern Saharan Africa, African Sahel, South Africa, Middle East, southern India, Mexico, Brazil and the Amazon basin area [18].

The Amazon region spans territories of France (French Guyana) and eight countries (Bolivia, Brazil, Colombia, Ecuador, Guyana, Peru, Suriname and Venezuela) [19]. The Amazon biome covers 49.5% of the Brazilian territory [20], which holds around 60% of the Amazon rainforest [21]. The Brazilian Amazon occupies the states of Acre (AC), Amapá (AP), Amazonas (AM), Roraima (RR), Pará (PA), Rondônia (RO), and parts of the state of Tocantins (TO), encompassing 93.2% of the Northern Region. The rainforest also comprises parts of the states of Maranhão (MA) and Mato Grosso (MT) in the Northeast and Central-West regions, respectively [20].

The North and Northeast regions comprise 52% and 26%, respectively, of the Brazilian scorpion fauna [22]. The twenty-six states of Brazil and the Federal District are geopolitically divided into five macroregions: North, Northeast, Central-West, Southeast, and South [23]. More than 70 scorpion species were identified in the North region [7, 22, 24], of which 48 species were recorded in the state of Amazonas [25-27]. The geographic distribution of the 28 species of the genus *Tityus* will be shown in the section “3.1. Ecological aspects of scorpions from the Brazilian Amazon region”.

The increasing number of envenomings and deaths caused by scorpions, mainly in urban centers, has been a public health problem in Brazil for years [28, 29]. The accelerated process of urbanization and the lack of basic infrastructure (such as water, electricity, sewage treatment, and regular collection of garbage) have provided conditions for the proliferation of opportunistic and invasive scorpions of high ecological plasticity, such as *T. stigmurus* and *T. serrulatus* [28, 29]. The later species is responsible for the most serious envenomings and deaths in Brazil [30].

Scorpion stings are worrisome because they represent the majority of incidents and can be of high severity, which makes it difficult for sanitary agencies to manage cases [31].

Since 2004, the number of scorpion envenoming cases in Brazil has exceeded those caused by snakebites [30], and in 2018, these figures were 156,833 against 28,946 [32]. However, some Brazilian states provide updated notification records with a time lag, sometimes one or two years after the report [33]. Scorpion stings were the most frequent accidents caused by venomous terrestrial animals in Brazil (46%), and responsible for almost 31% of deaths from 2000 to 2018, when compared to accidents caused by snakes, spiders, bees and caterpillars [34, 35]. It is noteworthy that the number of cases of scorpion accidents increased about 70% from 2016 to 2018 [35].

In Brazil, since the beginning of the 21^st^ century, the highest severity of scorpion envenoming has been notified in the Northern region [33, 36-38]. However, it is important to highlight that not all parts of the Brazilian Amazon have records about the species of scorpions involved in accidents.

Although the North region comprises 52% of the Brazilian scorpion fauna [22], only the venom of *T. obscurus* has been extensively studied [39-48]. *T. apiacas, T. metuendus, T. silvestris* and *T. strandi* are also species of medical interest in this region [36, 37, 49-52], but there are no studies on the biochemical and molecular characterization of the toxins present in these venoms. 

According to the clinical manifestations, the scorpion envenoming is classified into mild (local pain and paresthesia), moderate (intense local pain associated with one or more systemic manifestations) or severe (cardiac and hemodynamic changes, cardiogenic shock and pulmonary edema that can evolve to death) [11, 36]. Local manifestations are classified as mild symptoms and represent about 87% of the recorded scorpionism cases [29, 30]. Because of the pain, patients may experience nausea, agitation, and mild tachycardia, which will disappear after local treatment. In these cases, the patient is kept under observation for at least 6 hours, and any worsening of the symptoms requires hospitalization for clinical management [30]. Among the severe manifestations are countless vomiting episodes, profuse sweating, tachypnea, increased blood pressure, tachycardia or bradycardia, and symptoms compatible with acute congestive heart failure due to increased vascular resistance and acute lung edema [11, 29, 53]. Patients presenting systemic manifestations of scorpion envenoming are managed with symptomatic treatment, antivenom serum, and cardiorespiratory support [30].

From 2013 to 2017, about 83% of deaths resulting from scorpionism occurred within 48 hours after the sting [29]. The severity of scorpion envenoming is related to cardiac and hemodynamic changes, with cardiogenic shock and pulmonary edema contributing to the main causes of death [30]. Most victims of lethal scorpion stings die from cardiac or respiratory failure [11].

The notified scorpionism cases analyzed during the period from 2001 to 2012 revealed the highest severity of scorpion envenoming in the northern Brazil [33]. Only 60% of the envenomings were asymptomatic or mild in the North region (against 80-90% in the rest of the country). Moderate and severe symptoms accounted, respectively, for 35% and 4% in the North region, against, respectively, less than 15% and 2.5 % in the other four macroregions [33]. This discrepancy is due to the *scarcity* of studies on the epidemiology and venom characterization of the main scorpion species causing accidents and clinical manifestations. Such lack of data is due to, among other factors, incorrect species identification, inaccurate diagnosis and limited accessibility to antivenom treatment. These aspects make scorpionism a relevant public health problem, especially in the Brazilian Amazon region [54, 55].

The specific treatment for moderate and severe scorpion stings, in Brazil, consists of antivenoms produced against *T. serrulatus* venom, species that does not belong to the Brazilian Amazon region [56]. A case report showing that severe symptoms caused by *T. silvestris* sting were refractory to anti-*Tityus* antivenom [50] illustrates the need to develop new antivenoms or improve the effectiveness of those available. Despite the territorial extension of the North region, the specific clinical care and professional support to taxonomically distinguish venomous animals that are life-threatening is usually carried out in the capitals of the Brazilian states of Amazonas and Pará, respectively, Manaus (03°05′S 60°02′W) and Belém (1°26′S 48°29′W) [9, 50]. The severity of the systemic effects caused by *Tityus* species depends on the venom composition and the patient's clinical condition [29]. Therefore, it is essential to identify and characterize the components within the scorpion venom to produce more effective antivenoms.

It is important to highlight that many animal venoms, including those from non-health threatening scorpions, may provide diagnostic tools, experimental molecules to validate postulated therapeutic targets, drug libraries and prototypes for the design of drugs and therapeutic agents [57]. Furthermore, studies on the biology of scorpions can contribute to anticipate the risk of envenoming and reduce the severity of accidents [58].

In view of this scenario, this manuscript systematically reviews the reported symptoms and epidemiology of envenoming, ecological aspects of scorpions, and characterization of venoms and toxins to access the gaps that need to be filled in the knowledge of scorpion species of medical importance that occur in the Brazilian Amazon.

## Methods

A systematic review was carried out following the rules and guidelines of the Preferred Reporting Items for Systematic Reviews and Meta-Analyses (PRISMA) statement [59].

### Search strategy and data collection

An electronic search was performed in the following general databases: Web of Science all databases (Zoological Record, Web of Science Core Collection, Current Contents Connect, and Scielo Citation Index), Scopus, Virtual Health Library (VHL, which hosts Medline and the Latin American and Caribbean Center on Health Sciences Informational - LILACS), Embase, Pubmed and Cochrane Library. An electronic search was also performed on the Brazilian Digital Library of Theses and Dissertations (BDTD) (https://bdtd.ibict.br/vufind/). The search string used in these bases was “Amazon* AND *scorpi* AND NOT (pseudoscorpi* OR pseudoescorpi* OR scorpioides)”. Concerning Embase, “$scorpi*” was used instead of “*scorpi*”.

An electronic search was performed on the gray literature base Opengrey (https://opengrey.eu), and on the preprint servers BioRxiv (https://www.biorxiv.org/) and MedRxiv (https://www.medrxiv.org/), using the keywords “Amazon scorpion”.

Manual search and some alternative sources, such as reference lists from other studies and reviews, were consulted to ensure the inclusion of relevant articles. 

No restrictions on date (from 1864 to 2020), language or status (abstract or full text) of the publication were used. The searches were carried out on December 11^th^, 2020.

### Criteria

The study selection process was carried out by two independent reviewers, and any disagreement was solved by consensus. The selection process was verified by a third reviewer, ensuring the specificity and quality of the process. The selection of studies was carried out in two stages. In the first stage, the titles and abstracts of the references identified through the search strategy. Articles that did not meet the inclusion criteria or met the exclusion criteria were removed and the potentially eligible studies were pre-selected. In the second stage, the full text evaluation of the pre-selected studies was carried out to confirm eligibility. The selection process was carried out through the StArt (State of the Art through Systematic Review) tool (http://lapes.dc.ufscar.br/tools/start_tool) [60].

**Figure 1.**
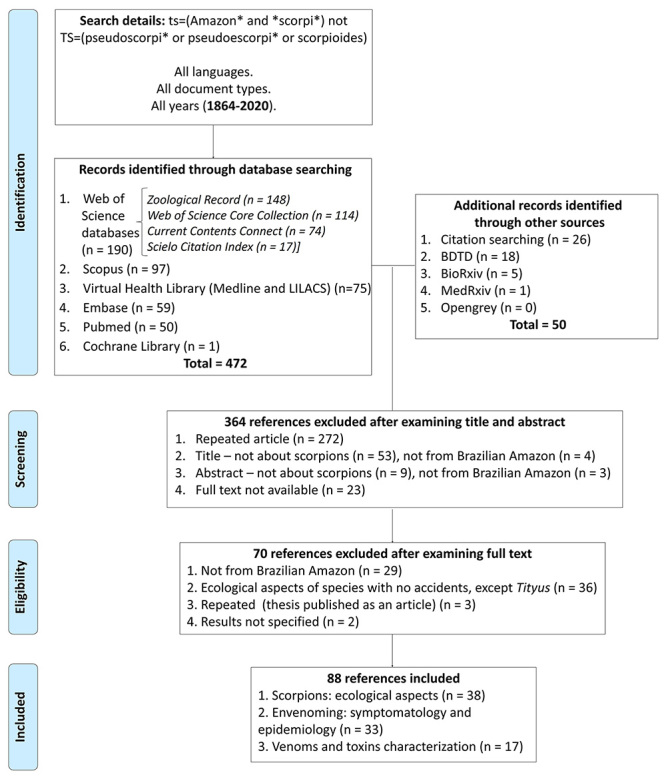
PRISMA flowchart showing the total number of records identified and filtered at each stage of the selection process obtained from the literature search of a systematic review on Amazonian scorpions.

When the searches from all the bibliographic databases were combined, 522 records were obtained (Fig. 1). A total of 272 repeated records were found in the combined dataset, leaving 250 papers to be examined for inclusion/exclusion criteria. All articles related to Amazonian scorpions were included. The articles were excluded from the final analysis if they met any of the following exclusion criteria: (*a*) articles not about scorpions, (*b*) concerning non-Amazonian scorpions, (*c*) full text not available, (*d*) not from Brazilian Amazon, (*e*) ecological aspects of species with no accidents (except *Tityus*), and (*f*) repeated (thesis published as an article).

After title and abstract screening, 181 relevant papers were obtained for full paper screening. Three theses were removed because they were published as papers and 23 papers could not be obtained. During full text screening, a total of 88 studies didn’t meet the exclusion criteria. These remaining articles were divided into three groups, considering the following inclusion criteria: (*a*) ecological aspects of scorpions, (*b*) symptomatology and epidemiology of envenoming, or (*c*) venoms and toxins characterization, as shown in the PRISMA flowchart in Figure 1.

## Results

### Ecological aspects of scorpions from the Brazilian Amazon region

**Figure 2.**
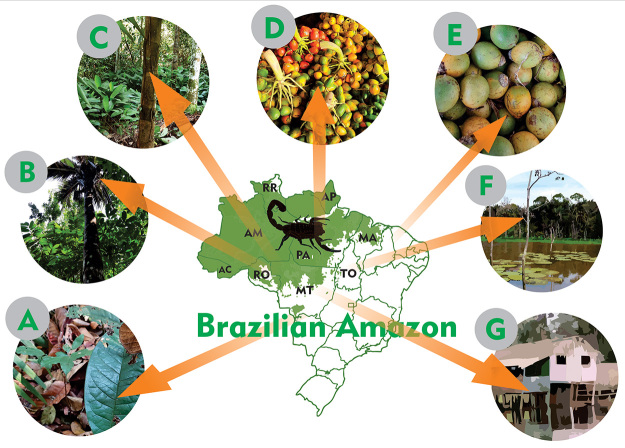
Places where scorpion envenoming are more likely to occur in the Brazilian Amazon. **(A)** Forest leaf litter. **(B)** Amazon canopy. **(C)** Arecaceae trunks. During the harvesting and handling of fruits, such as **(D)** *Bactris* spp. and **(E)** *Astrocaryum* spp. **(F)** Floodable forests (known as *igapó* and *várzea* in Brazil). **(G)** Houses in rural and urban areas. Figure by Nícolas da Silva Garcia.

Scorpions native from Amazon basin are equilibrium species that depend on natural conditions [61, 62]. Such animals inhabit stable environments and demonstrate habitat and microhabitat specificity [61]. Populations of *Tityus* scorpions, in the Brazilian Amazon, are most abundant in *terra firme* forest (non-flooded area) (Table 1), which comprises the largest Amazonian vegetation cover [61]. Places where scorpion envenoming are more likely to occur in the Brazilian Amazon are shown in Figure 2 (A-G).

**Table 1.** Ecological, morphological and reproductive aspects of scorpions from the Brazilian Amazon.

Species	Morphology	Body size (mm)	Habitat	Micro-habitat	Brood size	Ref.
*Brotheas amazonicus* (Chactidae), Lourenço, 1988	General coloration dark brown and reddish telson. Carapace moderately granular; tergites I to IV punctuate; VII moderately granular. Pectines with 8 to 11 teeth.	60-70	*Terra firme* forest (non-flooded zone)	These scorpions hide mainly under decaying trunks. During the night, they are detected near burrows in the leaf litter (~20 cm depth). Specimens are found in the urban region or near rural settlements.	8-21	[7, 15, 64, 65]
*Rhopalurus laticauda* (Buthidae), Thorell, 1876 (synonym *R. amazonicus* Lourenço, 1986 ; *R. crassicauda* Lourenço, 2002)	Yellowish-brown coloration with metasomal segment V and dark telson. Exhibits phenotypic plasticity in size and the intensity of infuscation on the carapace, tergites, metasoma, and pedipalps.	50	Amazonian savannah	Under rocks, wood barks and fallen trunks.	19	[66, 67]
*T*. (*Archaeotityus*) *bastosi*Lourenço, 1984	Dark yellow background densely covered with dark reddish-brown variegated spots. Dorsolateral keels of metasomal segments I to IV with a very strong spinoid posterior granule.	33-41	*Terra firme* forest	Specimens hide under leaves and wood barks in the leaf litter.	36	[68]
*T.* (*Archaeotityus*) *clathratus* Kosch, 1844	Dentate margins of pedipalp-chela fingers composed of 12 to 14 oblique rows of granules.	25-30	*Terra firme* forest	Leaf litter.	8-18	[25]
*T*. (*Archaeotityus*) *grahami*Lourenço, 2012	Yellowish to reddish-yellow with brown to dark brown spots over the body and appendages. Metasomal segment V granulated; vesicle with ventral and lateral carinae.	25-37	*Terra firme* forest	These scorpions can be found in the leaf litter or in tree trunks.	-	[26]
*T.* (*Archaeotityus*) *maranhensis* Lourenço et al. 2006	Coloration yellowish to reddish-yellow. Chelicerae with residual spots, and sternites pigmented, dorsal carinae of metasomal segments I to IV with spinoid. granules, internal carinae of patella with marked granules.	32-34	*Terra firme* forest	Leaf litter.	-	[26]
*T.* (*Archaeotityus*) *mattogrossensis* Boreli, 1901	Yellowish with dark pigmentation on the body. Dentate margins of pedipalp-tibia fingers composed of 15/16 oblique rows of granules.	30-36	Open vegetation	In termite mounds or on Arecaceae.	12	[68, 69]
*T*. (*Archaeotityus*) *silvestris* Pocock, 1897	Yellowish with dark spots over the body. Dorsolateral keels of metasomal segments I to IV without a spinoid posterior.	25-45	*Terra firme* forest and floodable forest (*igapó* and *várzea*)	They are detected in leaf litter, in the trunk of Arecaceae or among fruits in the Amazonian canopy. Specimens are found inside houses in the urban and rural areas.	5-14	[62, 65, 70-72]
*T.* (*Atreus) anori* Lourenço, Rossi and Wilmé, 2019	Reddish-brown to dark brown. I) Chela with an inconspicuous scalloping of the proximal dentate margin of fixed finger in male; III) better marked carinae on mesosoma; IV) very weak chetotaxy on pedipalps; V) sternites III and V with a white triangular zone on posterior edge.	88	Floodable forest	They hide under tree trunks or in the Amazonian canopy.	-	[25]
*T.* (*Atreus*) *apiacas* Lourenço, 2002	General coloration reddish-brown, with some yellowish zones on the sternites. Metasomal segments I to V blackish-brown, with 10-8-8-6(8)-5 darker carinae. Dentate margins of pedipalp-chela fingers with 16 oblique rows of granules.	75-100	*Terra firme* forest.	These scorpions can be detected in the leaf litter or in tree trunks. They are found in rural communities, in fruit and vegetable plantations.	-	[51, 73]
*T.* (*Atreus*) *dinizi* Lourenço, 1997	Blackish, but with some pale regions on the sternites. Basal middle lamellae of pectines of males slightly dilated. Dentate margins of pedipalp-chela fingers with 16 rows of granules; pectines with 20 teeth.	85-100	Floodable forest	They hide under tree trunks or in the Amazonian canopy.	-	[68, 73, 74]
*T.* (*Atreus*) *elizabethae* Lourenço and Ramos, 2004	Reddish to dark reddish overall. Metasomal segments with 10-10-10-8(7)-5 carinae.	72	Amazonian savannah	They are found in the leaf litter or in tree trunks.	-	[75]
*T.* (*Atreus*) *generaltheophiloi* Lourenço, 2017	Blackish-brown to dark blackish, particularly on the legs and pedipalps. Metasomal segments I to V, with 10-8-8-8-5 carinae, crenulated. Basal tooth on fixed finger of chelicera has a particular trifid morphology.	70.5	*Terra firme* forest (Mountain forest at altitudes of 600 m)	These scorpions can be detected in the leaf litter or in tree trunks.	-	[24]
*T.* (*Atreus*) *matthieseni* Pinto da Rocha and Lourenço, 2000	General coloration brownish with some dark spots over the body and pedipalps; legs brownish with yellowish spots. Metasomal segments I to V and telson uniformly blackish-brown; with 10-10-8-8-5 carinae.	75-85	*Terra firme* forest	They are found under wood bark in the leaf litter or in palm leaf sheaths.	-	[76]
*T. (Atreus) metuendus* Pocock, 1897	Blackish-brown to blackish. Metasomal segments I to III blackish-brown, IV and V blackish; ventral keels of metasomal segments I to IV parallel.	80-90	*Terra firme* forest and floodable forest	They are detected in leaf litter, in the trunk of Arecaceae or among fruits in the Amazonian canopy. Specimens are found in the urban region or near rural settlements.	25-35	[7, 65, 67, 68, 73, 77, 78]
*T.* (*Atreus*) *neblina* Lourenço, 2008	General coloration reddish-yellow to reddish-brown overall. Metasomal segments with 10-8-8-8(7)-5 carinae. Dorsal carinae of metasomal segments III and IV have 1 to 3 spinoid granules.	45-52	*Terra firme* forest (Mountain forest at altitudes of 850-2200 m)	Scorpions can be detected in the leaf litter or in tree trunks.	-	[76]
*T.* (*Atreus*) *obscurus* Gervais, 1843 (synonym: *T. cambridgei* Pocock, 1897; *T. paraensis* Kraepelin, 1896)	Blackish. Metasomal segments I to V and telson uniformly blackish; with 10-10-8-8-5 carinae. Trichobothria^*^ et and est on fixed finger of chela only slightly proximal; Est in the chela not basal to Esb.	75-100	*Terra firme* forest and floodable forest (*igapó* and *várzea*)	Specimens are found in leaf litter, in the trunk of Arecaceae, on Amazonian canopy or near rural settlements.	15-25	[15, 69, 71, 79]
*T.* (*Atreus*) *tucurui* Lourenço, 1988	Coloration blackish-brown dentate margins of pedipalp-chela fingers with 16 oblique rows of granules; pectines with 20 to 21 teeth.	85-100	*Terra firme* forest	These arthropods hide in the vegetation or in the palm leaf sheaths.	-	[68, 73]
*T*. (*Atreus*) *unus*Pinto da Rocha and Lourenço, 2000	Blackish-brown. Trichobothria^*^ et and est on fixed finger of chela proximal; Est in the chela basal to Esb.	70-80	*Terra firme* forest	They are detected in the leaf litter or in tree trunks.	-	[76]
*T.* (*Brazilotityus*) *adisi* Lourenço & Pézier, 2002	Yellow-variegated pigmentation. The dentate margins of the pedipalp-tibia fingers are composed of 12/13 oblique rows of granules.	20	Floodable forest (*igapó*)	Hide in tree trunks or in the Amazonian canopy.	-	[76]
*T.* (*Brazilotityus*) *lokiae* Lourenço, 2005	Coloration yellowish with variegated brown spots over the body and appendages. Subaculear tubercle very and spinoid.	27	Floodable forest (*igapó*)	Hide in tree trunks or in the Amazonian canopy.	-	[76]
*T.* (*Brazilotityus*) *rionegrensis* Lourenço, 2006	General color variegated from pale yellow to reddish. Fixed and movable finger cutting edge of pedipalp hands with 10-11 longitudinal series of granules.	30	Floodable forest	Amazonian canopy.	-	[76]
*T.* (*Tityus*) *canopensis* Lourenço & Pézier, 2002	Generally pale yellow without spots or pigmented regions on the body and its appendages.	10.3	Floodable forest (*igapó*)	Hide in tree trunks or in the Amazonian canopy.	-	[76]
*T.* (*Tityus*) *carvalhoi*	Coloration reddish-brown; pedipalps without spots; dentate margins of pedipalp-chela fingers composed of 16 oblique rows of denticles; pectines with 23 to 24 teeth.	45-50	*Terra firme*	-	-	[76]
*T. (Tityus) gasci* Lourenço, 1981	General coloration yellowish or with diffuse spots; basal middle lamellae of female pectines at the same level as the anterior tooth.	63	*Terra firme.*	Leaf litter.	-	[76]
*T.* (*Tityus*) *marajoensis* Lourenço & Silva, 2007	Coloration yellow-wish, with carapace and tergites reddish-brown to brown, much darker than appendages. The tergites are divided by a yellow longitudinal strip.	49	Marajó island (0°58′S 49°34′W)	-	-	[80]
*T.* (*Tityus) nelsoni* Lourenço, 2005	Yellowish to reddish yellow, without any marked spots over the carapace. Basal middle lamellae of female pectines not dilated.	55-60	*Terra firme.*	-	-	[76]
*T.* (*Tityus*) *raquelae* Lourenço, 1988	Coloration yellowish without spots; pedipalp-chela fingers composed of 15 oblique rows of denticles; pectines with 17 to 18 teeth.	55-60	*Terra firme* forest.	These animals hide in tree trunks or in palm leaf sheaths.	-	[65, 78, 81]
*T. (Tityus*) *strandi* Werner, 1939	General coloration yellowish-brown; tergites with confluent pale brown spots; basal middle lamellae of female pectines with only half of its surface at the same level as the anterior tooth.	50-70	*Terra firme* forest.	They hide in the vegetation, mainly Arecaceae. They are found in the urban region or near rural settlements.	12	[7, 65, 68]
*T.* (*Tityus*) *sylviae* Lourenço, 2005	Coloration yellowish to reddish yellow, with confluent dark spots over the carapace the tergites. Fixed finger with 15 and chela movable finger with 16 oblique rows of granulates.	45-50	*Terra firme* forest	Specimens can be found in fallen trunks.	-	[76]

Subgenus is shown in parentheses: *Genus (subgenus) species*; -: no information; Trichobothria^*^: trichobothrial notations (et, est, Est, Esb) used to distinguish the two species; Ref.: reference.

In the states of Pará and Amazonas, Brazil, which together have a large area of *terra firme* forest, scorpions such as, respectively, *T. obscurus* and *T. metuendus*, can be found in leaf litter (dead plant material, including leaves, bark, needles, and twigs, that has fallen to the ground) (Fig. 2A), in palm leaf sheaths, on the canopy (which may be over 100 feet (30 m) above the ground) (Fig. 2B) or in Arecaceae trunks (Fig. 2C). Among the species found in the Brazilian Amazon region, twenty-eight species of the genus *Tityus* are reported in Table 1.

In different areas of the rainforest, it is possible to find four scorpion subgenera: *Archaeotityus* Lourenço, 2006, *Atreus* Gervais, 1843, *Tityus* C. L. Koch, 1836 (subgenus) and *Brazilotityus* Lourenço, 2006 [63]. All these subgenera are notified in the Brazilian Amazon and their ecological, reproductive and morphological aspects are displayed in Table 1.

Scorpions *Tityus* can live about two meters from the ground, in the insertion of palm leaves or inside bromeliads suspended in trees over 30 meters above the ground [82]. Specimens of the subgenera *Atreus* and *Brazilotityus* can be found in micro-habitats at a height of up to 40 m from the ground in some regions with dense forest, suggesting that they can reproduce on the Amazon canopy [65, 69]. The availability of hiding places for arboreal scorpions is huge in the Amazon. It is estimated that 16,000 species of trees occur in the Amazon rainforest [83]. However, scorpions dangerous to humans, such as *T. metuendus, T. silvestris* and *T. obscurus*, are found mainly in Arecaceae (previously designated as Palmae) [69, 84], the most common botanical family in the Amazon region [85] that comprises 38 genera and 270 species [86]. Palm trees produce a great variety of fruits that serve as daily food for the population of the Amazon basin [87]. 

*Tityus* specimens are usually found in contact with the trunk of palm trees (Fig. 2C), including *Bactris* and *Astrocaryum* [70, 84]. *T. silvestris* can be detected in the trunk of *Bactris gasipaes* Kunth or may prefer to hide among their fruits produced at a height of about 20 m [29, 70, 87]. Palms of genera *Mauritia, Bactris, Euterpe* and *Astrocaryum* are micro-habitats for harmful scorpions (Table 1). Some scorpions, such as *T. obscurus, T. strandi, T. silvestris, T. apiacas* and *T. metuendus*, can hide among fruits, such as “açaí” (*Euterpe* spp.), “pupunha” (*Bactris* spp.) (Fig. 2D) and “tucumã” (*Astrocaryum* spp.) (Fig. 2E) [38, 69, 70]. The palm fruits usually come from dense forests or from rural plantations in the Amazon, which are geographical areas of greatest contact with venomous animals. Arecaceae species usually develop their fruits in clusters [87], which serve as shelter for scorpions and other arthropods. The harvest of these fruits and especially the handling in open markets or at home, can increase the risk of stings [38, 70].

In *igapó* and *várzea* forests (flooding zones) (Fig. 2F), invertebrates, including scorpions (*Tityus*), migrate vertically to the tree trunks or canopy before the annual flood period [65, 67, 88]. This seasonal event prevents the terrestrial activity of several venomous animals and the risk of human envenoming can decrease in these areas, usually for 5 to 7 months [65, 88]. Furthermore, the ecological conditions of these forests during the flood period lead to a decrease in animal hunting, cultivation of plants, fruit collections and, consequently, contact with venomous animals that hide in leaf litter and in tree trunks. However, several groups of vertebrates and invertebrates, such as scorpions, can move to human communities that are established a few meters from rivers. In the Brazilian Amazon, *Atreus* scorpions are the species with the greatest presence in flooded forest (*igapó*) (Table 1). These animals can be found even inside the houses or in the surroundings of the houses (Fig. 2G), favoring the risk of accidents [54] during all the year, since the seasonal incidence of scorpion stings is steady in North region [33].

**Figure 3.**
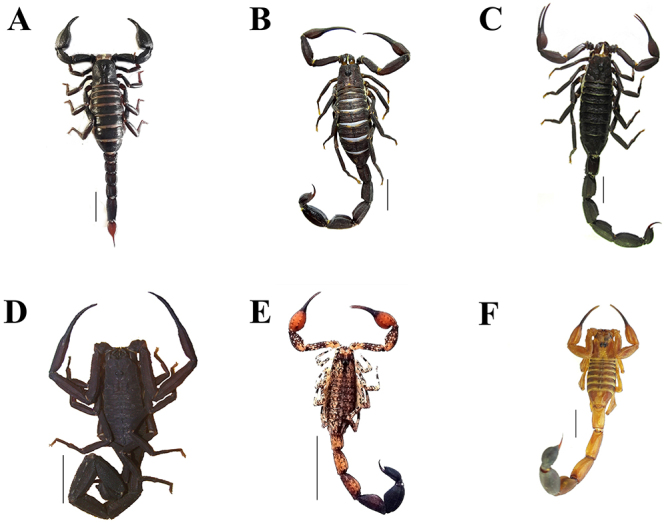
Scorpion species of the Brazilian Amazon region: **(A)** *Brotheas amazonicus*; **(B)** *Tityus apiacas*; **(C)** *Tityus metuendus*; **(D)** *Tityus obscurus* (photo by Pedro P. O. Pardal, reprinted with permission); **(E)** *Tityus silvestris* (photo by Bruno R. R. Almeida, reprinted with permission); **(F)** *Tityus strandi*. Scale bar (A-G) = 1 cm.

Chactidae family concentrates a large number of species in the Northern region of Brazil living on *terra firme* forest, mainly under fallen trunks and burrows in the ground [64]. Populations of *B. amazonicus* Lourenço, 1988 (Fig. 3A), a member of the Chactidae family, can be found mainly in the region of Manaus [64]. The specimen of *B. amazonicus* shown in Figure 3A was found between roots of *Mauritia flexuosa* L. f. palm, popularly known as *buriti*, in the city of Manaus, Amazonas state capital, Brazil. Fewer and less severe accidents caused by the scorpions *Ananteris* sp. (Buthidae) and *B. amazonicus* have been registered in the Brazilian Amazon region [89]. Although the venom of *B. amazonicus* has no medical relevance, it caused rapid paralyzing effects in adult crickets *Gryllus assimilis* and in larvae of tenebrioid beetles *Zophobas morio*. In larvae, this effect persisted for 24 hours when higher doses of venom (20 µg) were used [90]. The first report of a caecilian amphibian (Siphonopidae: *Microcaecilia* sp.) being preyed upon by a scorpion, whose genus was identified as *Brotheas*, was recently published [91].

Local manifestations have also been registered after stings of *Rhopalurus laticauda* Thorell, 1876 (synonym *R. amazonicus* Lourenço, 1986 and *R. crassicauda* Lourenço, 2002) (Buthidae) [92], which is endemic to Amazon savannah [66]. The venom and major β-neurotoxin Rc1 from *R. laticauda* show pro-inflammatory activities *in vitro* and a nociceptive response *in vivo* [93].

### The geographic distribution of the genus *Tityus* in the Brazilian Amazon

The geographic distribution of a total of 28 species belonging to four subgenera of the genus *Tityus* in the Brazilian Amazon is shown in Table 2, including some species found on Amazon canopy that have no reported envenoming cases yet [25, 26, 54] (Table 1).

Table 2 indicates a higher number of *Tityus* species in the state of Amazonas (n = 19), followed by the state of Pará (n = 8), and lower in the states of Roraima (n = 4), Mato Grosso (n = 4), Acre (n = 3), Rondônia (n = 2) and Amapá (n = 2). In Maranhão, it was considered only the record of *T. maranhensis*, which was found in an area in the state with Amazonian vegetation. In Tocantins, the occurrence of *T. (Atreus)* sp. was registered [109]; however, the specimens of the collected arachnids were not identified. The region of Manaus stands out for the highest number of species of *Tityus* notified (n = 7). In the Eastern Amazon, comprised by the states of Pará, Maranhão, Amapá, Tocantins and Mato Grosso, *T. obcurus* was recorded in 39 municipalities (Table 2). However, this species is found mainly in the state of Pará, where it is widely distributed and more related to moderate and severe cases of scorpionism [99, 102, 110] (Tables 2 and 3). *T. silvestris* and *T. metuendus* have a wide geographic reach in the Northern region (Table 2), as proposed previously by other studies [7, 31, 69]. *T. strandi*, which has been recorded in the state of Amazonas, also occurs in the states of Mato Grosso and Pará [81]. On the other hand, there are no new geographic occurrences for *T. apiacas*. *Tityus* scorpions with no confirmed records for the Brazilian Amazon are *T. thelyacanthus*, *T. magnimanus* and *T. rufofuscus* [75, 97, 111]. Although cases of accidents with *Atreus* can evolve into severe manifestations, the toxic potential of these arachnids is still unknown. Despite the geographic reach of the genus *Tityus* being underestimated in the Brazilian Amazon, the spatial distribution of scorpions of medical importance showed in Table 2 can assist health agencies in the prevention and prediction of moderate and severe accidents. On the other hand, surveillance should be extended to species still with no notification of stings (Table 2).

**Table 2.** Geographic distribution of scorpions of the genus *Tityus* in the Brazilian Amazon.

Species of medical importance	Locations (State: Municipality)	References
*T. apiacas*	**Amazonas**: Apuí. **Mato Grosso**: Apiacás	[74, 94, 95]
*T. metuendus*	**Amazonas**: Silves, Rio Preto da Eva, Autazes, Manacapuru, Itacoatiara, Novo Airão, Manaquiri, Itapiranga, Careiro da Várzea, Beruri, Iranduba, Tabatinga, Parintins, Presidente Figueiredo, Manaus, Novo Aripuanã. **Acre**: Rio Branco, Mâncio Lima, Senador Guiomard, Cruzeiro do Sul, Brasiléia, Xapuri **Pará**: Juriti, Óbidos, Santarém. **Mato Grosso**: Alta Floresta. **Rondônia**: Guajará-Mirim, Porto Velho. **Roraima**: Amajari.	[72, 74, 77, 94, 96-99]
*T. obscurus*	**Pará**: Belém, Santarém, Barcarena, Ananindeua, Juruti, Almeirim, Jacundá, Bujaru, Benevides, Ourém, Ipixuna do Pará, Colares do Pará, Alenquer, Santa Izabel do Pará, São Francisco do Pará, Primavera, São Félix do Xingu, Acará, Conceição do Araguaia, Breves, Melgaço, Parauapebas, Tomé-açu, Salvaterra, Tucuruí, Belterra, Uruará, Afuá, Santa Bárbara do Pará, Santo Antônio do Tauá, Rurópolis, Abaetetuba, Novo Progresso, Traírão, Aveiro, Traquateua, Castanhal, Óbidos. **Amapá**: Serra do Navio.	[9, 70, 95-97, 100-102]
*T. silvestris*	**Amazonas**: São Paulo de Olivença, Novo Airão, Benjamin Constant, Tabatinga, Tefé, Maraã, Manaus, Presidente Figueiredo, Rio Preto da Eva, Iranduba, Manacapuru, Itacoatiara. **Pará**: Juriti, Belém, Santarém, Tucuruí, Bragança, Acará, São João de Pirabas, Parauapebas, Melgaço, Almeirim, Altamira, Itaituba, Benevides, Ananindeua, Mocajuba, Óbidos, Baião, Rurópolis, Santo Antônio do Tauá. **Amapá**: Serra do Navio. **Mato Grosso**: Aripuanã, Cláudia. **Rondônia**: Ji-paraná, Guajará-Mirim. **Acre**: Senador Guiomard, Rio branco.	[72, 74, 94, 95, 97, 100, 103, 104]
*T. strandi*	**Amazonas**: Manacapuru, Beruri, Barcelos, Uarini, Coari, Manaus. **Pará**: Melgaço, Altamira, Vitória do Xingu, Santarém, Rurópolis, Tucuruí, Juruti, Monte alegre. **Mato Grosso**: Cotriguaçu, Aripuanã.	[74, 95, 97, 100, 103, 105]
**Species with no recorded accidents**	**Locations (State: Municipality)**	**References**
*T. adisi*	**Amazonas**: Tarumã Mirim, Manaus.	[65]
*T. anori*	**Amazonas**: Anori.	[25]
*T. bastosi*	**Amazonas**: Tefé, São Paulo de Olivença, Tabatinga.	[74, 95, 97]
*T. canopensis*	**Amazonas**: Tarumã Mirim, Manaus.	[65]
*T. carvalhoi*	**Pará**: Piracuruca. **Mato Grosso** (no municipality specified)	[97, 99]
*T. clathratus*	**Roraima**: Amajari, Boa Vista.	[95, 104]
*T. dinizi*	**Amazonas**: Novo Airão.	[74, 94]
*T. elizabethae*	**Roraima**: Pacaraima.	[73, 75]
*T. gasci*	**Amazonas**: Urucará. **Acre**: Mâncio Lima, Rio Branco, Sena Madureira, Senador Guiomard. **Pará** (no municipality specified)	[74, 106]
*T. generaltheophiloi*	**Roraima**: Parque Nacional da Serra da Mocidade	[24]
*T. grahami*	**Amazonas**: Santa Isabel do Rio Negro, Barcelos.	[26, 95]
*T. lokiae*	**Amazonas**: Tarumã Mirim, Manaus.	[65, 67]
*T. marajoensis*	**Pará**: Marajó island (0°58′S 49°34′W).	[80]
*T. maranhensis*	**Maranhão**: Caxias (Inhamum Ecological Reserve).	[107]
*T. matthieseni*	**Amazonas**: Manicoré.	[74, 76]
*T. mattogrossensis*	**Mato Grosso**: Cuiabá, Alto Araguaia.	[104]
*T. neblina*	**Amazonas**: Parque Nacional do Pico da Neblina (0°48'01"N 66°0'25"O).	[73]
*T. nelsoni*	**Amazonas**: São Gabriel da Cachoeira.	[108]
*T*. *raquelae*	**Amazonas**: Manaus, Rio Preto da Eva, Presidente Figueiredo.	[74, 81]
*T. rionegrensis*	**Amazonas**: São Gabriel da Cachoeira.	[54]
*T. sylviae*	**Amazonas**: Barcelos.	[108]
*T. tucurui*	**Pará**: Baião, Tucuruí.	[95, 96, 100, 103]
*T. unus*	**Amazonas**: Santa Isabel do Rio Negro.	[74, 76, 95]

**Table 3.** Symptomatology of victims stung by scorpions of medical interest from the Brazilian Amazon region and *in vivo* effects of venoms on experimental animals.

Species	Symptomatology of envenoming	Ref.
*T. apiacas*	Immediate local pain, electric shock sensation (Apuí, AM), local erythema, local edema, sweating, vomiting, diarrhea, pallor, tremors, myosis and agitation.	[51]
*T. metuendus*	Cause human envenoming. **Humans:** local pain, hyperemia, paresthesia, edema, tachypnea, lethargy, psychomotor agitation, mental confusion, myoclonia, sweating, sialorrhea, dyspnea, nausea, vomiting and tachycardia. **Mice:** restlessness, piloerection, sialorrhea, hyperactivity, respiratory difficulties, partial paralysis of limbs, exophthalmos, loss of equilibrium, convulsions and death.	[36, 52] [113] [98]
*T. obscurus*	**Humans: Belém (PA)** - Local manifestations: local and radiating pain, paresthesia, edema, erythema, sweating, piloerection and burning. **Santarém region (PA)** - Neurological manifestations: electric shock sensation, dysdiadochokinesia, dysmetria, dysarthria, dyslalia, nausea and vomiting, compatible with acute cerebellar dysfunction. **Rats:** hemorrhagic patches in the lung parenchyma and no pulmonary edema; decrease in general activity; no changes in the occurrence and intensity of induced convulsions; no hippocampal neuronal loss. **Mice:** edematogenic and moderate nociceptive activity; decreased locomotion, breathing difficulty, piloerection, palpebral ptosis and excessive oral and nasal secretions. The effects began 30 min after venom injection and persisted for about 3 h, while the respiratory changes persisted for 6 h.	[9, 49, 102, 114] [115] [115]
*T. silvestris*	Local manifestations: local pain, paresthesia and edema. Systemic manifestations: nausea, vomiting, somnolence, malaise, dyspnea, tachycardia, headache, myoclonia, hyper/hypotension, hypothermia, abdominal pain and generalized muscle spasms.	[50, 70, 113]
*T. strandi*	Local pain, paresthesia, erythema, edema, dysesthesia with a tingling sensation and electric shock sensation (Santarém, PA) of variable extension.	[37, 105]

Ref.: reference. The atypical sensation of electric shock registered in cases of scorpionism in the Brazilian Amazon is underlined.

### Scorpion envenoming in the Brazilian Amazon - symptoms and epidemiology

The genus *Tityus* is of medical importance [30], and four species (*T. serrulatus*, *T. bahiensis*, *T. stigmurus* and *T. obscurus*) are capable of causing serious accidents in Brazil [89, 112]. The incidence of envenoming with scorpion stings is increasing in the Brazilian Amazon, mainly due to the species *T. metuendus*, *T. apiacas*, *T. silvestris* and *T. obscurus* [54]. Recently, the first three cases of envenoming by *T. strandi* were recorded in Santarém (2°25′48″S 54°43′12″W), state of Pará [37]. These five scorpion species (*T. apiacas, T. metuendus, T. silvestris, T. obscurus* and *T. strandi*) of medical relevance in the Brazilian Amazon region [36, 37, 49-52] are reported in Table 3.

**Figure 4:**
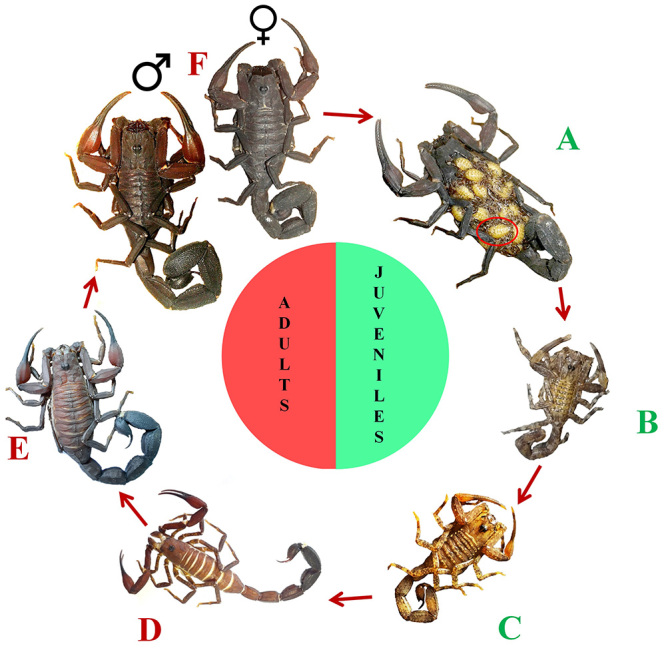
Ontogenetic development of *T. metuendus*. **(A)** A 3-day old newborn (red circle). **(B, C)** Juveniles of 20 and 75 days old. **(D, E)** Adults aged between 390 and 515 days. **(F)** Male and female adults of undetermined age. *T. metuendus* adults are responsible for many cases of envenoming in the Brazilian Amazon. Photos of panels A, B and F by Francisco José Ramos Prestes, reprinted with permission.

Many patients, who seek public health care in the Brazilian Amazon after being envenomed, report they were stung by a black scorpion [49, 114]. *T. (Atreus) obscurus* Gervais, 1843, is known as the black scorpion of the Amazon [112]. However, the animal color may not contribute to the elucidation of the cases. Depending on the geographical area, different dark-colored species, such as *T. anori, T. dinizi, T. generaltheophiloi, T. unus, T. matthieseni, T. elizabethae, T. tucurui, T. apiacas, T. metuendus* and *T. obscurus*, may be related to human envenoming. These large (65 to 110 mm total length) Amazonian scorpions belong to the subgenus *Atreus* and are very similar when adults (Fig. 3B-D, Table 1). The similarity between *Atreus* scorpions in the Brazilian Amazon region is complex (Table 1) and can induce misidentifications [99]. Throughout the ontogenetic development of *T. metuendus*, the species exhibits a remarkable difference in the coloration of juveniles and adults (Fig. 4A-F) and the main responsible for human envenoming are adult specimens (Fig. 4D-F) [113]. During this stage of life, *T. metuendus* can be widely detected in *terra firme* forest and in rural and urban areas of the Amazon [64, 66]. Juveniles scorpions of *T. metuendus* (Fig. 4B-C) may be mistakenly confirmed as *T. silvestris*, *T. grahami* (morphological data in Table 1), or as juveniles of *T. obcurus* [99]. Besides *Atreus* scorpions, the Amazon encompasses complexes models of polymorphism observed for species of *Tityus*, such as *T. silvestris*, *T. gasci* and *T. bastosi* [13, 104, 116], which may hinder species identification. The correct distinction of scorpions involved in accidents helps in the diagnosis and in the prediction of serious complications, depending on the causative species [36, 113]. Surprisingly, in several cases of scorpion envenoming, the animals responsible for the stings are not identified in the Brazilian Amazon region (as will be shown later in Table 4) [36, 117-119]. In the Northern region, the identification of venomous animals is usually carried out in Manaus and Belém, respectively, the capitals of the Brazilian states of Amazonas and Pará [9, 50]. Other cities lack a professional qualification structure to taxonomically distinguish venomous animals that are dangerous to humans. It is also worth highlighting that out of 369 presumed scorpion stings treated in Manaus between June 2014 and December 2019, about 61% (225 cases) had no identified causative agent [113]. Scorpion species capable of causing moderate and severe human envenoming accidents in the Brazilian Amazon region are shown in Figure 3 (B-F). Some listed species possess populations widely distributed in the Amazon basin [7, 62]. *T. metuendus* is a monomorphic species and can use parthenogenesis for producing offspring [120], without needing a sexual partner. Such mechanism of reproduction contributes to the high dispersion of animals and incidence of scorpion stings [61].

**Table 4.** Epidemiological aspects of scorpionism in the Brazilian Amazon region.

Scorpion species/Animal description	Region	Number of cases/Period/Time of day and year	Gender/Age of the victims (years)	Sting location	Time between the accident and the medical assistance	Main signs and symptoms	Clinical manifestations	Treatment administered	Ref.
***T. obscurus* and *T. silvestris***	Belém (Pará, Brazil) (1°26′S 48°29′W)	61 Jan. to Dec.1996	54.1% female Age range 0-7 (21.4%) 7-55 (65.3%) ˃ 55 (13.3%)	Upper limbs - hand (40.9%); neck (3.3%); elbow (1.6%) Lower limbs - foot (31.2%); thigh (14.7%)	-	**Local symptoms** - pain (83.9%), edema (47.5%), hiperemia (36%), paresthesia (6.6%) **Systemic symptoms** - vomiting (13%), tachycardia (3.2%), nausea (3.2%), somnolence (3.2%), paleness (3.2%), shock (1.6%), sweating (1.6%)	Mild (86.9%) Moderate (11.4%), Severe (1.7%)	Specific antivenom serum (26.2%)	[110]
***T. obscurus*** (8.3%) Designation - “*lacrau*” (a synonym in Portuguese for the word scorpion) (87.5%); scorpion (4.2%), did not know (8.3%). Description - black (5.6%), black and large (59.7%), yellow (1.4%), and did not know inform (33.3%). Took the animal (8.3%).	Municipal Hospital of Santarém - Santarém (2°25′48″S 54°43′12″W) (94.4%), Belterra (02°38′09″S 54°56′13″W) (4.2%), Prainha (1°48′0″S 53°28′48″W) (1.4%) (Pará, Brazil)	72 Feb. 2000 to Feb. 2001 Morning (40.3%) Afternoon (41.6%) 19-6 h (18.1%) The highest incidences occurred in Mar. (18.1%), Aug. (18.1%) and Apr. (11.1%)	83.3% male Age range (median 33.6 ± 18.3 years) 2-14 (13%) 84.1%) > 65 (2.8%)	Upper limbs (51.5%) - hands (41.1%) Lower limbs (43.1%) - foot (38.9%) Other body parts (5.4%).	Time ranged from 30 min to 14 h, with an average of 4.6 ± 3.2 h.	**Local symptoms** (91.7%) - paresthesia (79.2%), pain (52.8%), edema (26.4%). **Neurological signs** (97.2%) - myoclonia (93%), electric shock sensation (88.9%); dysmetria (86.1%), dysarthria (80.6%), ataxia (70.8%)	Moderate (76.4%), with no serious cases	Specific antivenom serum was not administered in 32.7% of the moderate cases (unavailable). Antiscorpionic serum (63.6%) and antiaracnidic serum (3.7%), with an average of 3.5 ± 0.8 ampoules. Patients discharged cured - within 24 hours (98.6%), in 3 days (1.4%).	[49]
***T. obscurus*** Specimens were taken by the patients and identified.	Eastern and western areas of the state of Pará (Brazil)	48 Jan. 2008 to July 2011	Eastern: 50% male Western: 64.3% male Age range Eastern: < 5 (2.9%) 11.8%) > 15 (85.3%) Western: < 5 (7.1%) 6-14 (7.1%) > 15 (85.8%)	Eastern: upper limbs (70.6%); lower limbs (23.5%); other parts (5.9%) Western: upper limbs (57.2%); lower limbs (35.7%); other parts (7.1%)	Eastern: < 1 h (64.7%) 2-3 h (14.7%) > 3 h (20.6%) Western: < 1 h (57.1%) 2-3 h (14.3%) > 3 h (28.6%)	**Eastern:** Local symptoms - pain (88.2%), radiating pain (5.9%), paresthesia (47.1%); systemic manifestations - sweating (5.9%), somnolence (2.9%) **Western:** local symptoms - pain (100%), radiating pain (64.3%), paresthesia (85.7%); systemic manifestations - sweating (35.7%), somnolence (28.6%), tremors (35.7%), agitation (28.6%), electric shock sensation (50%), myoclonus (64.3%), dysarthria (42.8%)	Eastern: Level 1 (76.5%) Level 2 (17.6%) Dry sting (5.9%) Western: Level 1 (35.7%) Level 2 (64.3%)	Eastern: Painkillers (76.5%) Antivenom (17.6%) Western: Painkillers (35.7%) Antivenom (64.3%)	[9]
-	State of Amazonas - highest incidence in Apuí (7°11′49″S 59°53′27″W) and Rio Preto da Eva (2°41′56″S 59°42′0″W))	2,120 (56.6% from rural areas; 38.7% work-related accidents; 72.4% farmer/fisher) Jan. 2007 to Dec. 2014	63.9% male 0-10 (14.8%) 11-20 (18.6%) 21-30 (19.7%) 31-40 (18.1%) 41-50 (14.8%) 51-60 (8.2%) > 61 (5.7%)	Upper limbs (47.9%); lower limbs (46.5%), body (3.3%), head (2.3%)	< 3 h (69.6%) 4-6 h (17.1%) 7-12 h (6.9%) 13-24 h (3.4%) > 24 h (2.8%)	-	Mild (68.6%) Moderate (26.8%), Severe (4.6%) Death (0.3%)	-	[36]
-	State of Pará (Brazil)	13,453 2007 to 2014	65.8% male Age ranged from 1 to > 60 years old 1-14 (20.2%) 15-19 (9.5%) 20-39 (38.6%) 40-59 (23.9%) >60 (7.8%)	-	0-1 h (37.6%) 1-3 h (30.4%) 3-6 h (17.2%) 6-12 h (8.2%) > 12 h (6.6%)	-	Mild (55.8%) Moderate (39.1%) Severe (51%)	-	[124]
-	Cruzeiro do Sul (Acre, Brazil) (7°37′51″S 72°40′12″W)	164 2012 to 2017	68.2% male Age range 0-10 (13.5%) 11-20 (10.1%) 21-30 (15.2) 31-40 (25.4%) 41-50 (10.1%) 51-60 (6.4%) ˃ 60 (18.6%)	Upper limbs - forearm (1.3%), upper arm (1.3%), hand (55.7%); Lower limbs - foot (35.3%), lower leg (4.0%), thigh (0.6%)	0-1 h (45.1%) 1-3 h (27.0%) 3-6 h (15.2%) 6-12 h (6.9%) 12-24 h (2.0%) ˃24 h (3.4%)	**Local symptoms (100%)** - pain (81.7%), edema (66.8%); **Systemic manifestations (5.4%)** - neuroparalytics (4.7%); Vomiting/diarrhea (2.0%)	Mild (67.3%) Moderate (25%) Severe (7.6%)	Serum therapy (68.9%)	[117]
***T. obscurus*** Black	Santarém (Pará, Brazil) (2°25′48″S 54°43′12″W)	28 2013 to 2014	60.7% male Age range 1 - 10 (7.1%) 11 - 20 (7.1%) 21 - 30 (32.1%) 31 - 40 (14.3%) 41 - 50 (17.9%) 51 - 60 (7.1%) 61 - 70 (14.3%)	Upper limbs - hand; lower limbs foot	-	**Neurological manifestations** - electric shock sensation, ataxia, dysarthria, dysmetria	-	No antivenom available (1%), antivenom administered 10 hours after the accident (1%)	[38]

-: no information. Ref.: reference.

Epidemiological data indicate that *Atreus* scorpions are responsible for most *Tityus* envenoming and lethality reports in the Brazilian Amazon [9, 49, 51, 52, 113]. However, the number of species involved in the scorpionism in the Brazilian Amazon may be greater than reported in the literature. This macroregion encompasses 52% of the Brazilian scorpion fauna [22], where new species are continually being discovered [24-26]. However, displacement challenges faced by the envenomed victim, such as rivers on the way, dirt roads, areas with poor road access and long distances to reach the local health units, can result in under-notification [54]. Despite geographical barriers, it is important to raise awareness of scorpionism, including prevention, pathophysiological effects and treatment, especially in the Brazilian Amazon region, where a large number of scorpion-endemic venoms has not been studied yet and whose potential for lethality is unknown. For instance, the upper Rio Negro (0°58'26"S 62°55'32"W) region (state of Amazonas, Brazil) concentrate scorpion species with high degree of endemism and biology poorly known [121]. This is an area with complex floristic composition, which can hinder human access for sample collections and studies of scorpion fauna [108]. Although *T. matthieseni* may be related to some envenoming cases in the state of Amazonas [27], no clinical description has been reported for this species [54].

Human populations living in close contact with the forest, such as indigenous people, rubber tappers and rural workers, are the most susceptible to accidents with venomous animals [56, 118]. In 2017, the incidence coefficient (per 100,000 workers) due to work accidents involving scorpions in the rural areas, forest and waters was higher in municipalities in the North region, mainly in the states of Amazonas, Pará, Amapá and Tocantins, than in the other four Brazilian macroregions [122]. About 10% of tappers and 14% of Amerindians from the state of Acre were stung by scorpions at least once in their lifetime [118]. Scorpion stings occurred in the forest are the most underreported accidents and the species that caused the envenoming is generally not identified [118]. Nevertheless, a technical effort to elucidate the dynamics of accidents in the forest and urban-forestry areas indicated that the stings occur most often during the day, especially in the workdays. However, night accidents (18.1% of the cases) also occurred, when scorpions tend to be more active and imperceptible to people [49]. Many scorpion stings have occurred in the comfort of home in many parts of the Brazilian Amazon. From 1998 to 2005, 52% of accidents in the region of Belém, state of Pará, occurred at home and the most affected members were hands and feet [119]. Infrastructure problems, such as the garbage collection and disposal, sanitation (sewage and storm drain systems), are responsible for scorpions’ dispersion in large Brazilian cities [29, 123]. Although the introduction of these venomous animals into human space in the Amazon region should be investigated, the harvesting of fruits and vegetables in rural areas and the exploitation of wood in native forest probably contribute to the dispersion of scorpions to urban areas [70]. People who live especially in rural settlements in the Amazon rainforest often climb trees several meters high to harvest fruits. This practice increases the risk of stings, mostly on the victims’ hands and feet, by arboreal scorpions that hide in trunks and among bunches of fruits [11, 13]. The first case of a male adult stung by *T. serrulatus*, a non-native species to Pará, while unloading bananas at the supply center in Belém (Amazon region) was recently reported. The banana bunches from Bahia (Northeast region) were transported by truck to Belém. If the species were introduced to the Northern region, it could cause ecological disturbances and become a public health problem [101].

Mild and moderate symptoms are frequently recorded after scorpion envenoming in the Brazilian Amazon [33, 36, 113, 117]. Intense pain, paresthesia, edema and erythema are the most reported local symptoms [9, 113]. Depending on the scorpion species, neurological manifestations stand out among the systemic effects [49, 114]. Epidemiological aspects of scorpionism in the Brazilian Amazon region are shown in Table 4.

Although cardiac and hemodynamic changes may culminate in fatal outcomes from cardiogenic shock and pulmonary edema, complementary exams that aid in diagnosis, such as biochemical tests, electrocardiogram, chest radiography and echocardiography [30], are rarely performed.

The prognosis depends on the time between a sting and the patient’s arrival at the hospital/appropriate treatment [29]. The time before arrival at hospital was later in North region when compared to the other four Brazilian regions. Only about 35% of the patients in North region were admitted at the hospital during the first hour after the scorpion envenoming (against 50% in the other four macroregions) [33]. Pharmacokinetic assays showed that biodistribution of *Androctonus australis hector* scorpion venom from the injection site to the tissues is within 15 min [125], which reinforces the need for early health care.

Severe symptoms of scorpionism in the Brazilian Amazon region (Table 3) mainly affects people living in precarious conditions [126]. Most of these people are farmers, rubber tappers, traders, domestic workers and hunters who work directly with the resources extracted from the forest [118]. From an epidemiological point of view, these occupations are more vulnerable to scorpion envenoming [118].

Most victims of lethal scorpion stings die from cardiac or respiratory failure [11] and a direct relationship among the inflammatory process, neuronal activation, neurotransmitter storm, cardiac dysfunction, and mortality induced by scorpion venom was recently established [127]. Mild or severe scorpion envenomings, such as those caused by, respectively, *R. laticauda* and *T. serrulatus*, can activate the canonical nuclear factor-kappa B (NF-(B) pathway that mediates inflammatory responses [93, 128] and is supposed to be one important mechanism of enhancing the immune responses after scorpion envenoming [129].

In the Brazilian Amazon region, mortality is also associated with delayed access to care [130], lower literacy levels and income than in the Southeast region [130], due to geographical barriers and long distances to reach local health units [54], the delay in receiving immunotherapy [33, 36, 54] and sometimes the specific antivenom is not available (Table 4) [49]. Pharmacokinetic studies showed that venom concentrations were maximal at 15 min in the kidney and liver, and at 30 min in serum, lung, heart and spleen, after subcutaneous injection of *T. serrulatus* venom [131]. The antivenom therapy is frequently administered in patients in the western region of the state of Pará, where more than 97% of the patients had systemic and neurological signs (Table 4) [49].

People living in the Amazon region have their daily lives altered by accidents with several venomous animals, which can even compromise the livelihood of their families [50]. *Tityus* stings can cause a person to stay from a few hours to several days in the hospital. For instance, a man spent 9 days in care at the Manaus Tropical Medicine Foundation after being stung by *T. silvestris* [50].

Populations of *T. metuendus* are abundant in different parts of the Amazon basin, and adult specimens (Fig. 4D-F) are the main responsible for human envenoming, especially in the region of Manaus. The venom of this arachnid causes paresthesia, nausea, blurred vision, respiratory failure, myoclonia, edema, pain, sialorrhea and tachycardia [113]. Several patients with similar or more severe symptoms face the difficulty of locating hospitals in the Amazon with specific antivenom and clinical support with experience in this type of emergency.

Strikingly, harmful scorpions which belong to different subgenera, such as *T. obscurus* and *T. strandi* (Table 1), depending on the Amazon region where they live [126], can cause cerebellar-muscular changes in the envenomed victims. The reported neurological manifestations are not observed in patients stung by scorpions in other regions of Brazil [38, 49], where typical autonomic disturbances prevail, such as those caused by *T. serrulatus* [132]. 

The reported neurological symptoms caused by *T. obscurus* (synonymous species *T. cambridgei* and *T. paraensis*) [133], in the region of Santarém, state of Pará, such as myoclonus, fasciculations and a sensation of shock, evidenced a regional symptomatology [49, 89]. A clinical, mitochondrial (16S rRNA), morphometric and proteomic study with populations of *T. obscurus* indicated that distinct lineages occur in the eastern and western regions of the state of Pará [126]. In the western region (Santarém), one of the symptoms most reported by patients is the sensation of electric shock [49, 114], usually reported when *T. obscurus* is involved in the accidents [49, 114]. This symptom is also reported after envenomings caused by *T. apiacas* and *T. strandi* (Table 3) [37, 51]. Significant toxic variations in these venoms may be peculiar to some Amazon regions [38, 49, 51]. Since several populations of *Tityus* live in microregions in the dense forest [25, 27], assessing the toxicity of these species’ venoms can be complex. Clinical manifestations compatible with neuromuscular/somatosensory dysfunction and few manifestations compatible with adrenergic/cholinergic stimulation were also reported after *T. strandi* stings [37]. Three sudden fatal cases caused by *T. obscurus* stings in Guyana's remote jungle areas over a 12-month period indicate the potential for gravity caused by local populations of this species [134].

In the Amazon region, many victims of scorpion accidents needed immunotherapy (Table 4) and the number of cases requiring antivenom therapy is growing in the last years [51, 113, 114]. It is important to highlight that Brazilian scorpion antivenom is produced by immunizing horses with *T. serrulatus* antigen [56]. The literature reports a clinical case of *T. silvestris* envenoming refractory to the antiscorpionic serum produced against *T. serrulatus*. A 39-year-old patient, envenomed by *T. silvestris* in the urban area of Manaus, showed cerebellar-muscular changes (usually observed after accidents with *T. obscurus*) and required treatment with benzodiazepines, in addition to supportive therapy with hydantoins and antihistamines [50].

A study comparing the transcriptomic-proteomic profiles of the venoms from *T. serrulatus* and *T. obscurus* revealed that differences at primary sequence may reflect in different epitopes for the same protein classes in these two allopatric species, resulting in the poor recognition of *T. obscurus* venom by the Brazilian scorpion antivenom [47]. Furthermore, variation in toxicity due to the diversity of *T. obscurus* venom in different areas of the Amazon has been suggested [102] and most of the 320 NDBPs detected by a peptide profile from *T. obscurus* venom do not correspond to any known toxin [135]. Similarly, eight sequenced peptides, among 201 molecular species from 800 to 17,000 Da detected in the venom from the Amazonian scorpion *B. amazonicus*, showed no similarity degree with any known molecule [136]. This remarkable number of unknown toxins from Amazonian scorpions highlights the need to characterize these venoms for the development of more effective therapies.

Furthermore, the available Brazilian scorpion and arachnid antivenoms were not able to recognize *R. laticauda* venom and its fractions (with exception of hyaluronidase) [93]. These antivenoms were also not able to shorten the intensity and duration of the neurological manifestations in patients stung by *T. apiacas* or *T. obscurus* [113]. On the other hand, phage display technique allowed the isolation of scFv from a human library of antibodies against Ts1. This antibody fragment specific for Ts1 toxin from *T. serrulatus* also recognized toxins from the scorpions *T. packyurus* and *T. obscurus* from the Amazonian region [137]. The design of efficient serotherapies is challenged by the structural and antigenic polymorphisms reported in the α-toxin family [138]. For instance, *T. obscurus* and *T. serrulatus* venoms have toxins with distinct epitopes for the same protein classes [47]. 

To compare reactivity from medically important *Tityus* populations inhabiting Brazil, Colombia, Costa Rica, Ecuador, Panama, Trinidad and Tobago, and Venezuela against commercial antivenoms from Brazil, Venezuela, and Mexico, *in vivo* cross-reactivity studies and molecular assays, including MALDI-TOF mass spectrometry, cDNA sequencing, competitive ELISA, immunoblotting, and phylogenetic analyses were performed [139]. Based on venom composition and immunochemical criteria, *Tityus* spp. fauna inhabiting the Caribbean, Lower Central America (LCA) and South America was grouped into four venom antigenic regions. Species inhabiting Region I (LCA/Colombia/Amazonia) produce venoms that were not significantly reactive against available antivenoms [139]. In view of this scenario, further studies are needed to identify and characterize compounds from Amazonian scorpion venoms to improve the design of efficient antivenoms.

### Characterization of venoms and toxins of scorpions from the Brazilian Amazon region

Old World scorpion genera of Buthidae, including *Androctonus* and *Leiurus*, have very potent neurotoxins specific for mammalian or insect Na^+^ channels, whereas New World scorpion genera, such as *Centruroides* and *Tityus*, have potent toxins acting on both mammalian and insect channels [63]. Despite the dangerous, painful and fatal effects caused by scorpionism, therapeutic properties of scorpion venoms have been explored for thousands of years and several scorpion venom compounds may represent promising leads for the development of new pharmaceuticals [11, 57].

Scorpion toxins have been explored as antiangiogenic [140], insecticide [141], tumor binding [142], antithrombotic peptide [143], potential intranuclear delivery tool to target cancerous cells [144], tools to understand the mechanisms triggered in chronic pain [145], models to study the mechanisms involved in sterile inflammation [129], and to treat autoimmune diseases [57, 146, 147].

The *T. obscurus* venom was firstly reported in 1998 and its first potassium channel toxin (Tc1) was characterized in 2000 [39]. Up to now, there are 48 and 33 transcripts from *T. obscurus* that have similarities with known, respectively, sodium and potassium channel toxins [47]. Among them, 9 NaTx and 1 KTx had been described before, of which 3 NaTx and 1KTx showed proteomic evidence [47]. There are three [39, 40] and four [41-43, 46, 48], respectively, potassium and sodium channel toxins from *T. obscurus* tested on electrophysiological assays (Table 5). A peptide profiling from *T. obscurus* venom detected 320 non-disulfide bridged peptides (NDBPs), which represents 5% of the crude venom, including thirteen novel peptides with inflammatory activities, identified as fragments of hypotensins, potassium channel toxins and the allergen 5 protein [135]. Interestingly, transcripts of phospholipase C were identified in species of *T. obscurus* and *T. serrulatus*, although no proteomic evidence has been detected. There is proteomic evidence of phospholipase A_2_ transcripts for *T. obscurus* venom only [47].

**Table 5:** Peptides and proteins identified in scorpion venoms from the Brazilian Amazon.

Scorpion	Toxin (synonym)	UniProt ID	MW (Da)	Sequence	Class	Tested channels/Characterization assays	Activity	Ref.
***Tityus. obscurus***	Tc1	P83243	2446	ACGSCRKKCKGSGKCINGRCKCY	α-KTx 13.1	Shaker B K^+^-channels	Blocks Shaker B K^+^-channels	[39]
***Tityus obscurus***	Tc30	P60210	3878	VFINVKCRGSKECLPACKAAVGKAAGKCMNGKCKCYP	α-KTx 4.4	Kv1.3 and Shaker B K^+^ (analog of the mammalian Kv1.1)	Blocks Shaker B( Kv1.1) and Kv1.3	[40]
	Tc32	P60211	3521	TGPQTTCQAAMCEAGCKGLGKSMESCQGDTCKCKA	α-KTx 18.1	Kv1.3 and Shaker B K^+^ (analog of the mammalian Kv1.1)	Blocks Shaker B( Kv1.1) and Kv1.3	
***Tityus obscurus***	To2 (Tc48a)	-	7310	DKDGYLMEGDGCMNGCLTRKASYCVDQCKEVGGKNGY...	NaTx	-	-	[41]
	To3 (Tc49a)	-	7141	KDGYLVGNDGCKYNCLTRPGHYCANECSRVKGAD...	NaTx	-	-	
	To1 (Tc49b)	-	7405	KKEGYLVGNDGCKYGCITRPHQYCVHECELKKGTDGYCAYWLACYCYNMPDWVKTWSSATNKCK	NaTx	Shaker B K^+^-channels; Na^+^ currents of granular cells	Changes Na^+^ currents of granular cells	
	To4 (Tc54)	-	7259	KDGYLMEYGGCKMSCLMKKGTFCAEECT...	NaTx	-	-	
*Brotheas amazonicus*	Venom peptide 1	P86341	978	IWSGIQGAF	*-*	-	-	[136]
	Venom peptide 2	P86340	1008	IWSGIQSAF	-	-	-	
	Venom peptide 3	P86344	1045	IGDIWSGIQG	-	-	-	
	Venom peptide 4	P86339	1087	IIDFIPQIE	-	-	-	
	Venom peptide 5	P86343	1192	FIGDIWSGIQG	-	-	-	
	Venom peptide 6	P86342	1249	GFIGDIWSGIQG	-	-	-	
	Venom peptide 7	P86338	1429	VAIRIIWSDIQD	-	-	-	
	Venom peptide 8	P86337	1449	ISDDIQSIIQGIF	-	-	-	
***Tityus obscurus***	Tc1	P83243	2446	ACGSCRKKCK*...*	KTx	-	-	[42]
	Tc27	P84676	4103	DEGPKSDCKP*...*	-	-	-	
	Tc29	P84677	4150	FNGAVXIW*...*	-	-	-	
	Tc30	P60210	3871	VFINVKCRGS*...*	KTx	-	-	
	Tc31	P84678	4304	CSTCLDKP*...*	-	-	-	
	Tc32	P60211	3521	TGPQTTCQAA*...*	-	-	-	
	Tc33	P84679	3807	ILNRCCNDDN*...*	-	-	-	
	Tc35	P84680	3926	TGPQTXXQAA*...*	-	-	-	
	Tc37	P84681	7265	TAIRKCNPRT*...*	-	-	-	
	Tc39	P84682	2744	DDDDLEGFSE*...*	-	-	-	
	Tc40 (~To13)^1^	P84683	7796	IKNGYPRDS*...*	-	-	-	
	Tc41 (~To14)^1^	P84684	7109	KDDYPVDTAK*...*	-	-	-	
	To6 (Tc43)	P84685	7266	LDGYPLSKNN*...*	-	-	-	
	Tc46	P84686	6032	KEGYLFGSRG*...*	-	-	-	
	To2 (Tc48a)	P60212	7318	NKDGYLMEGDGCKMGCLTRKASYCVDQCKEVGGKDGYCYAWLSCYCYNMPDSVEIWDSKNNKCGK	NaTx	Na^+^-channel	Changes Na^+^-channel	
	To3 (Tc48b)	P69213	7385	KDGYLVGNDG*...*	NaTx	-	-	
	To3 (Tc49a)	P69213	7152	KDGYLVGNDG*...*	NaTx	-	-	
	To1 (Tc49b)	P60214	7405	KKEGYLVGND*...*	NaTx	-	-	
	Tc50	P84688	7073	LDGYPLSKIN*...*	-	-	-	
	To4 (Tc54)	P60215	7253	KDGYLMEYGG*...*	NaTx	-	-	
	Tc56	P84689	7299	EKGKEILGKI*...*	-	-	-	
	Tc58	P84690	5504	KKFGGFLXXI*...*	-	-	-	
	Tc61 (~To8)^1^	P84691	7105	KEGYLLGSRG*...*	-	-	-	
	Tc64	P84692	7628	GLRQKVQSLV*...*	-	-	-	
	To5 (Tc66)	P84693	6935	SYSGYPVTQK*...*	-	-	-	
	Tc83	P84694	25402	NDQCLVIEIL*...*	-	-	-	
***Tityus obscurus***	To3 (Tc48b)	P69213	7385	KDGYLVGNDGCKYNCLTRPGHYCANECSRVKGKDGYCYAWMACYCYSMPDWVKTWSRSTNRCGR	α-toxin	Na^+^-currents of cultured rat pituitary GH3 cells	Changes Na^+^-currents of cultured rat pituitary GH3 cells	[43]
***Tityus obscurus***	To1 (Tc49b)	P60214	7405	MTRFVLFISCFFLIDMIVECKKEGYLVGNDGCKYGCITRPHQYCVHECELKKGTDGYCAYWLACYCYNMPDWVKTWSSATNKCKGK	All proteins shared sequence identity with NaTx.	- (proteomic)	-	[44]
	To2 (Tc48a)	P60212	7318	MIRFVLFISCFFLIGTVVECNKDGYLMEGDGCKMGCLTRKASYCVDQCKEVGGKDGYCYAWLSCYCYNMPDSVEIWDSKNNKCGKGK				
	To3 (Tc48b/Tc49a)	P69213	7385	MTRFVLFISCFFVIGMVVECKDGYLVGNDGCKYNCLTRPGHYCANECSRVKGKDGYCYAWMACYCYSMPDWVKTWSRSTNRCGRGK				
	To4 (Tc54)	P60215	7253	MTRFVLFISCFFLIGMIVECKDGYLMEYGGCKMSCLMKKGTFCAEECTRMKGKDGYCYAWLACYCYNMPDWVKIWNRATNKCGKRK				
	To5	P84693	6937	MKAIIFFIGCLMLIDLVAGSRSGYPVTQKGCVYSCFWGSNWWCNAECTALGGSSGYCAWPSCWCYSLPDNRNIWGSYPNNCGKK				
	To6 (Tc43)	P84685	7266	MSIFPIILALLLIGLDEGEALDGYPLSKNNYCKIYCPDEKVCKWSCKHRAGATNGKGDCINKGCYCYDVAPGTEMYPGRLPCNPY				
	To7 (Tc50)	P84688	7073	MSIFPIVLALLLIGLEETEALDGYPLSKINNCKIYCPDDDVCKWTCKHRAGATNGKGDCIWYGCYCYDVAPGTKMYPGSSPCYA				
	To8	H1ZZH7	7050*	MTRFVLFISCFFLIGMVVECKEGYLLGSRGCKMNCLTRPEKFCELECSLVGGENGYCAYWLACYCYNVPESVKLWESDTNECGKRK				
	To9	H1ZZH8	7155*	MNYSTLIAVASLLTAGTESKKDGYPVKEGDCAFPCGYDNEYCDKLCKERKADSGYCYWGNILCYCYGLPDKAAIKGYGRCRPGKK				
	To10	H1ZZH9	6940*	MNYSTLIAVASLLTAGTESKKDGYPVEGSCAFPCGYDNAYCDKLCKERKADSGYCYWVNILCYCYGLPDNAAIKGYGRCKPGKK				
	To11	H1ZZI0	7154*	MTRFVLFISCFFLIGMIVECKDGYLVGNDGCKYNCLTRPGHYCANECSRVKGKDGYCYAWMACYCYNMPNWVKTWSRATNKCGKRK				
	To12	H1ZZI1	7171*	MKGLILFICGFMMIGVILAKEGYPMDHEGCKFSCFIRPSGFCERYCKTHLSASTGYCAWPACYCYGVPANQKVWDYYNNKCGK				
	To13	H1ZZI2	8054*	MKTLFLIITSFILLEVEGIKNGYPRDSKGCTFECGQDAKHGDDYCDKMCKTTLKGEGGDCDFEYAECWCDNIPDTVVTWKNKEPKCKQI				
	To14	H1ZZI3	7953*	MNCLMLIFVVFLLAFGVECKKDDYPVDTAKRNCMLDCNVWDDEGYCDKFCKGRKADSGYCYKLKAACYCYGLPDDSPTKTSGRCNPNVR				
	To15	H1ZZI4	7195*	MKGIILLISCLMLIEVVVGGKEGYPLDSSGCKAGCFFGTNSWCNTECKRKSAAKGYCAWPSCYCYEFTDDSKIWNAKTNKCYK				
***Tityus obscurus***	Recombinant toxin Tc32	P60211	-	GSTGPQTTCQAAMCEAGCKGLGKSMESCQGDTCKCKA	α-KTx	Electrophysiological assays on periglomerular cells of olfactory bulb; three-dimensional (3D) solution structure determined by ^1^H NMR spectroscopy.	Blocks Kv1.1 and Kv1.3	[45]
***Tityus obscurus***	ToPI1	-	3807	DDCKDVCKARKGKCEFGICKC...	Serine peptidase inhibitor	Trypsin and chymotrypsin inhibitory assays; viability of the tumor cells HeLa (from human cervical cancer) and B16F10 (from murine melanoma) and non-tumor murine fibroblasts (NIH-3T3); EAG1, EAG2, hKv1.4, hKv1.1, hERG1, hERG2, hERG3 (5 uM)	Potent trypsin inhibitory activity; did not reduce the viability of tumor cells; no visible behavioral and/or physiological changes in mice; stable at the range of pH 3.0 to 9.0 even at 95 °C;	[148]
***Brotheas amazonicus***	-	-	-	-	Serine proteases	Proteolytic activity using SDS-PAGE	Proteolytic activity inhibited by PMSF	[149]
***Tityus obscurus***	ToAp1	A0A1D3IXR7	-	FIGMIPGLIGGLISAFK-NH_2_	AMP, NDBP subfamily 4	Antifungal activity against planktonic cells of *Candida* spp. and *Cryptococcus neoformans* and *Candida albicans* biofilms	Active against biofilm formation. Lower than 50% of hemolysis in all the tested concentrations. Active against *C. neoformans* and all *Candida* spp. (except for *C. glabrata*).	[150]
	ToAp2	A0A1D3IXJ5	-	MQFKKQLLVIFFAYFLVVNESEAFFGTLFKLGSKLIPGVMKLFSKKKERSLMKRELKNLYDPYQRSVEMERLLKELPLY	AMP, NDBP subfamily 3		Active against biofilm formation and all strains tested (MIC 3.12 to 200 μM). Hemolysis maintained at about 50%.	
	ToAP2S1	-	-	FFGTLFKLLSKLIPGLMKLFSKLLER-NH_2_	AMP, NDBP subfamily 3		Hemolysis percent higher than 50% in concentrations up to 25 μM. No antifungal activity.	
	ToAp3	-	-	FIGMIPGLIGGLISAIK-NH_2_	AMP, NDBP subfamily 4		Active against *C. neoformans* and all *Candida* spp. (except for *C. glabrata*).	
	ToAp4	-	-	FFSLIPSLIGGLVSAIK-NH2	AMP, NDBP subfamily 4		No antifungal activity.	
	NDBP-4.23	S6D3A7	-	FLGMIPGLIGGLISAFK-NH_2_	AMP, NDBP subfamily 4		Active against biofilm formation. Lower than 50% of hemolysis in all the tested concentrations.	
	ToAcP	A0A1D3IY23	-	EEDDLLGFSEEDLKAIKEHRAKNA-NH_2_	AMP, NDBP subfamily not designated		No antifungal activity.	
***Tityus obscurus***	To4 (Tc54)	P60215	-	KDGYLMEYGGCKMSCLMKKGTFCAEECTRMKGKDGYCYAWLACYCYNMPDWVKIWNRATNKC	β-toxin	hNav (1.1- 1.7)	Changes hNav 1.1, hNav 1.2 and hNav 1.4	[46]
***Tityus obscurus***	Transcripts with proteomic evidence	-	-	-	Angiotensin converting enzyme like Cysteine-rich protein, allergen V5/Tpx-1-related Endothelin-converting enzyme-like Hyaluronidase Metalloproteinase Serine and cysteine proteinases Phospholipase A_2_ KTx Proteinase inhibitors NaTx	-	-	[47]
***Tityus metuendus***	1 (24.41 min)	-	3927	TPFRYCNPRNCAKECQGRCKETTYCDEVCKCSGW	KTx	-	-	[98]
	2 (24.66 min)	-	1735	KVLAPAEEAPAEAPAAAE	bradykinin-potentiating peptide	-	-	
	3 (28.33 min)	-	4004	TAIGNCNPFTCDKECKTKGNKRGYCENYNCECSKW	-	Shaker-type ion-channels	-	
	4 (30.07 min)	-	7796/7767	KKNDYPVDTAKRNCMLDCNVWDDEGYCDNFCKGRKAESGYCYKLDAACYCYGLPDDSPTKTSGRCNPNV	NaTx	-	-	
	5	-	7318/7385	NKDGYLMEGDGCKMGCLTRKASYCVDQCKEVGGKDGYCYAWLSCYCYNMPDSVEIWDSKNNKCGK	NaTx	-	-	
	37.26 min	-	6961	KEGYLLGSRGCKMNCLTxPGNYCELECSLVGGxNG...	-	-	-	
	42.10 min	-	7153	SRRGYPVTQKGxVYSSFWGSN...	-	-	-	
	-	-	-	-	Angiotensin converting enzyme like Endothelin-converting enzyme-like Hyaluronidase Metalloproteinase	-	-	
***Tityus obscurus***	-	-	-	320 non-disulfide bond-containing peptides (NDBPs)	Fragments of hypotensins, KTx and the allergen 5 protein	-	-	[135]
***Tityus obscurus***	To1 (Tc49b)	P60214	-	KKEGYLVGNDGCKYGCITRPHQYCVHECELKKGTDGYCAYWLACYCYNMPDWVKTWSSATNKCK	β-toxin	hNa_V_ (1.1-1.7), BgNa_V_1, VdNa_V_1	Changes Na_V_ 1.3, Na_V_ 1.6, BgNa_V_1, VdNa_V_1	[48]
***Tityus obscurus***	Synthetic peptide ToAP2 (P6)	-	-	FFGTLFKLGSKLIPGVMKLFSKKKER	AMP, NDBP subfamily 3	Antiretroviral and cytotoxic activities	Active against simian immunodeficiency virus (SIV) replication in the HUT-78 cell line and in primary human leukocytes	[151]

(~To)^1^: N-terminal with which it shares identity (this work); *theoretical molecular mass reported (other molecular masses were determined experimentally); …: sequence not complete (N-terminal fragment); AMP: antimicrobial peptide; BgNa_v_: Na_v_ from the German cockroach *Blattella germanica*; EAG: *ether-à-go-go* channel; ^1^H NMR: proton nuclear magnetic resonance; hERG: the human *Ether-à-go-go*-related gene; hNa_v_: Na_v_ from humans; KTx: potassium channel toxin; K_v_: voltage-gated potassium channel; MIC: minimum inhibitory concentration; NaTx: sodium channel toxin; Na_v_: voltage-gated sodium channel; NDBP: non-disulfide-bridged peptide; Ref.: reference; VdNa_v_: Na_v_ from the mite *Varroa destructor*.

A comparative assay indicated that the dose of *T. obscurus* venom required to kill 50% (LD_50_) of mice (18-22 g) is 12.136 mg/kg, being classified as moderately toxic [15], whereas *B. amazonicus* (LD_50_ = 90.909 mg/kg) was practically nontoxic [15]. Other study reported the LD_50_ of 3.13 mg/kg and 0.99 mg/kg (intraperitoneal injection in mice (18-20g)) for *T. obscurus* and *T. serrulatus*, respectively [115]. It is noteworthy that the lethal dose (LD_50_) of the venoms of most scorpion species found in *igapó, várzea* and *terra firme* is still unknown.

Table 5 shows two studies on *B. amazonicus* venom, one study on the venom from *T. metuendus* and the remaining 14 reports (82%) are on *T. obscurus* venom. *B. amazonicus* venom degraded Aα and Bβ subunits of fibrinogen in sodium dodecyl sulphate-polyacrylamide gel electrophoresis (SDS-PAGE) and its proteolytic activity was inhibited in the presence of phenylmethylsulfonyl fluoride (PMSF), indicating the presence of serine proteases in the venom [149].

Electrophysiological assays using *T. metuendus* venom on seven sub-types of human voltage gated sodium channels (hNav1.1 to 1.7) revealed that it presents α- and β-scorpion toxins [98]. *In vivo* assays showed that *T. metuendus* venom was lethal to mice strain CD1 (25 g body weight) intraperitoneally injected with 200 μg and 300 μg of venom, respectively, within 80 min and 38 min after injection [98].

A recent proteomic analysis of the total soluble *T. metuendus* venom identified sodium and potassium channel toxins, metalloproteinases, endothelin and angiotensin-converting enzymes, bradykinin-potentiating peptide and hyaluronidases [98]. Arthropod venom hyaluronidases show potential medical and biotechnological applications [152]. The intranasal inoculation of hyaluronidase from *T. serrulatus* venom is a promising tool for pulmonary fibrosis treatment once it induces mononuclear increase in the bronchoalveolar space [153]. The use of hyaluronidase inhibitors can be used as a novel first-aid strategy in envenoming, since the enzyme has a key role in the scorpion venom spreading and biodistribution [154]. The toxic effects of scorpion envenoming were reduced by anti-hyaluronidase serum, which inhibited and delayed mouse death after subcutaneous injection of a lethal dose (13.2 (g) of *T. serrulatus* venom [155].

Scorpion venoms have a great diversity of molecules with potent and specific biological actions and, therefore, with possible biotechnological applications, justifying their study.

## Discussion

### Biases in this study

Systematic reviews must avoid publication biases as much as possible, including time lag, location, language, multiple publication, citation, and selective outcome reporting biases [156]. To avoid these publication biases, the following strategies were used in this manuscript: articles from 1864 to 2020 were retrieved; the searches were performed on 11 indexation databases (including grey literature, preprint databases and manual search); published and unpublished studies were searched; all languages available for the theme (English, French, Portuguese and Spanish), in the databases consulted, were included; theses were excluded if they were published as articles; all public (positive and negative) results available were presented in the text or in tables. The 88 reports included in this study were grouped as follows: 38 manuscripts about ecological aspects of scorpions; 33 reports about envenoming, including symptomatology, epidemiology and therapies; and 17 studies about venoms and toxins characterization.

Although none of the 88 reports included in this study discussed publication biases or any limitations of the study, we can point some of them. Concerning ecological information about the scorpion species studied, there is a lack of accurate geographical coordinates for different species, and the absence of a detailed description of the habitat and micro-habitat in which the species were found. These factors are impediments to conclusions about the biology of scorpions in the Brazilian Amazon. Regarding the cited epidemiological reports, they are observational studies which showed scorpion accidents refractory to antiscorpionic serum. Because correlation does not imply causation [157], an experimental randomized trial would be required in order to support a conclusion of cause and effect. On the basis of venoms and toxins characterization, proteomic reports identify several components, but lack functional characterization. And it is not possible to predict the function of these components, since most of them are novel molecules that do not share sequence identity with any reported primary sequence in databanks. Furthermore, there are some studies on the activity of enzymes that have not been isolated, impairing their structural characterization.

### Difficulties and limitations in the study

This review article was based on data and articles available on public databases in which information on complications in patients was very limited. The process of searching for information on accidents caused by venomous animals in the Brazilian Notifiable Diseases Information System (SINAN) database is a difficult task. Data are updated continuously in the website and some Brazilian states provide records with a lag, sometimes one or two years after the envenoming, which can result in variations in the presented data depending on the date the search was performed [33]. Much of the data relevant to epidemiology of scorpionism is not published and/or quite difficult to access or retrieve from public databases. It is noteworthy that data for the period 2016 to 2018 were retrieved from SINAN database by the Ministry of Health from May to September 2019 and are still subject to review (please see [158]). In addition, there are no data on accidents with scorpions in 2019 in the Ministry of Health website, showing that scorpionism is still a neglected public health problem in Brazil.

### Challenges related to research on scorpions and their toxins

The hardship in obtaining large amounts of venom and purified toxins is one obstacle faced when studying toxins from rare or small venomous species, such as scorpions [57]. For example, first venom extraction yields an average of 0.4 mg of venom from each scorpion and this amount is gradually reduced in the subsequent extractions [159]. In the Brazilian Amazon, the challenges to study the components from scorpion venoms begin initially with the collection of animals in a region of dense forest. Depending on the species, the capture of these arthropods may involve logistics that meet the conditions of the Amazon biome. Scorpions such as *T. metuendus, T. silvestris* and *T. obscurus* are abundant species in certain areas of the Brazilian Amazon, which offers opportunities for capture and reproduction in the laboratory for further assays. However, in an artificial environment, a large number of juvenile scorpions die, reducing the opportunity for more detailed experimental studies. In this case, the active collection of scorpions may present greater opportunities. The heterologous expression and chemical synthesis strategies have been used to overcome the limitation of amount of purified toxin.

After overcoming the challenge of obtaining enough quantity of the toxin, different cutting-edge technologies are used to integrate all data obtained through the “venomics” strategy, paving the way to explore novel compounds. The use of proteomics supported by mass spectrometry to explore animal venoms can elucidate the molecular mass of the toxin, fragments of its primary sequence, post translational modifications and their localization within the primary sequence [160]. However, the price of sophisticated instrumentation, maintenance of equipment and the need for qualified personal operating the instruments are usually costly. A difficulty faced by Brazilian researchers, mainly from the Northern and Northeast regions, is that the freight price and the waiting time for receiving an accessory or equipment may make the purchase of the product or maintenance of the equipment unfeasible, impairing the achievement of results. In the Northern region, few research groups study molecules originating from venomous animals, mainly due to the lack of specialists, “omic” tools, and financial support needed to discover the therapeutic value of venoms and achieve biotechnological development. A pioneering study of characterization of venom compounds from *T. metuendus* collected in the city of Manaus was made possible through a partnership abroad [98].

The next step involves experimental studies which mimics changes in the human body, since they have a key role for scientific evidence [161]. Animal experiments are needed to predict therapeutic doses, drug toxicity, pharmacokinetics, pharmacodynamics, and mechanistic information, but the use of mammalian models is being significantly reduced due to several reasons, including ethical and social dilemmas. In view of this scenario, the use of alternative models (such as brine shrimp, fruit fly, greater wax moth, roundworm, and zebra fish), development of non-animal methods and *in silico* modelling are required [162].

Regarding scorpionism, under-notification and low funding for investigating scorpion accidents result in the scarcity of data in the Brazilian Amazon. In addition to the lack of funding, epidemiological studies may be discontinued due to successive health programs that are ineffective in combating scorpionism.

### Research needs to fill knowledge gaps about scorpion species of medical importance in the Brazilian Amazon

In the Northern region lack a professional qualification structure to taxonomically distinguish venomous animals that are dangerous to humans. The identification of scorpions is usually carried out in Tropical Medicine Foundation Heitor Vieira Dourado (Fundação de Medicina Tropical Heitor Vieira Dourado, FMT-HVD), Manaus, Amazonas (03°05′S 60°02′W) and Laboratory of Medical Entomology and Venomous Animals, which is part of the Center of Tropical Medicine at the Federal University of Pará, Belém, Pará (1°26′S 48°29′W) [9, 50], but in some cases the scorpions were sent for identification or double check at the Special Laboratory of Ecology and Evolution at the Butantan Institute, São Paulo, Brazil (23°34′S 46°43′W) [37, 49, 51, 114].

Reported studies showed that some populations of *T. obscurus* in certain areas of the Brazilian Amazon may be more lethal, suggesting an intraspecific variability in the venom [9, 49], being notified a high recurrence of severe cases in the Santarém region [9, 29]. The atypical sensation of electric shock (Table 3) is reported after scorpion accidents in the states of Pará and Amazonas [30, 49]. In these last two states, new cases of scorpionism with neurological effects have been reported [37, 51, 113].

The epidemiology of scorpionism in the Brazilian Amazon is related to five species with varying colors and sizes (Fig. 3 B-F). These scorpions are responsible for envenomings that often evolve to a moderate and, in some cases, severe condition (Table 3). To the best of our knowledge, there are no studies showing the establishment of populations of scorpions *Tityus* in urban areas of the Brazilian Amazon, which is reported for populations of *T. serrulatus* and *T. stigmurus* in other regions of Brazil [61, 82].

In northern Brazil, forest regions host polymorphic populations of scorpions, mainly in the states of Amazonas and Pará [7, 103]. For example, the specimen of *T. silvestris* (Fig. 3E) is a representative of a population that occurs in the municipality of Santo Antônio do Tauá (01º09'S 48º08'00"W), state of Pará. However, it is not known if different polymorphic groups of *T. silvestris* in the Amazon have developed venoms capable of triggering clinical effects with regional symptoms. The sensation of electric shock reported by victims of *T. strandi* in Santarém (2°25′48″S 54°43′12″W), state of Pará [37], has not been reported so far in Cotriguaçu, Aripuanã, Manacapuru, Manaus, Beruri, Barcelos, Monte Alegre or in other municipalities where the species occurs (Table 2). The absence of these data can be related to the lack of knowledge about local scorpions, a small percentage (8.3%) of the envenomed victims take the scorpions to be identified [49] and a few health units are capable of registering and preserving the specimens brought by the victims.

Among the potential reasons for the under-notification of scorpion sting reports are the lack of scorpion antivenom (that leads people to doubt the usefulness of the surveillance system), lack of health professionals for operational issues, or patients being unable to access a health center for antivenom therapy. The improvement of systems of collecting data on scorpion accidents may improve the knowledge regarding scorpion envenoming, especially in areas with poor road access and geographical barriers.

Two studies showed that the available scorpion antivenoms have not been able to shorten the severity of neurological changes in patients stung by *T. apiacas*, *T. obscurus* or *T. silvestris*. Several studies worldwide have been performed to improve the manufacture and quality of available therapies. Next-generation antivenoms based on recombinant monoclonal antibodies and antibody fragments are expected to be more effective, safer, and cost-competitive therapies [11]. Studies on inhibitors, such as peptides, small molecules or bioactive components from plant sources, are also an interesting field to be explored as alternative approaches to improve the current limitations of serum therapy [154, 163-165].

Furthermore, there are few studies evaluating the immunogenic effect of Amazonian scorpion venoms on experimental animals (Table 3). These biological assays could contribute to the production of more effective therapeutic antibodies for the treatment of scorpion envenoming.

Experimental models and the knowledge about the scorpion toxins and biomarkers can be useful tools to help in early diagnosis in patients stung by scorpions, explain the clinical picture in humans, improve treatment and reduce fatal complications. Epidemiological and experimental studies are necessary to assess the relationship of an injury after an animal envenoming.

The venoms of scorpions from the Brazilian Amazon are still underexploited, lacking information on the chemical structure, physiological role and therapeutic application of their biologically active compounds. For example, there are eight peptides sequenced from *B. amazonicus* whose biological functions have not been evaluated. Although serine proteinase activity has been detected in this venom, the enzyme has not been isolated and no fragments of its sequence have been identified. Many compounds from *T. metuendus* and *T. obscurus* were identified through “omic” approaches, but no biological assays were performed (Table 5). Strikingly, 15 proteins from *T. obscurus* shared sequence identity with NaTx, but only four toxins (To1 to To4) were tested on electrophysiological assays. The study of venom components could increase the chances of discovering new molecules with great pharmaceutical potential and understanding the pathophysiological effects of the scorpion envenoming in the Brazilian Amazon.

## Conclusions

The critical assessment in this review showed that a meticulous study design is necessary to minimize bias during clinical and case reports about scorpion envenoming. We suggest that researchers become familiar with reporting guidelines such as the Strengthening the Reporting of Observational Studies in Epidemiology (STROBE), which are delineated to investigate the associations between an exposure and a health outcome [166].

More investment from the government and research foundations is required to raise awareness of the society on the importance of scorpion accident notification. Despite the highest severity of scorpion envenoming notified in the Northern region, there is a data scarcity on scorpionism in the Brazilian Amazon region, especially in remote areas.

*T. obscurus* is widely studied when compared to other Amazonian scorpions. However, data on its venom and its effects on human body are still little explored when compared to *T. serrulatus*. Furthermore, we still have dozens of scorpion species from the Brazilian Amazon with unexplored venoms. Although five species have been reported to be of medical importance in the northern region, the diversity of the scorpion fauna in this macroregion suggests that more species are involved in the scorpionism in the Brazilian Amazon.

Time, intellectual, technological and financial resources are also necessary to untangle the interaction of venom peptides with their target during the development of potential new engineered therapeutic molecules and more effective serum therapies. Antivenoms capable of neutralizing neurological changes caused by Amazonian scorpions are required, as well as mechanistic information about which and how the toxins present in the venoms of *T. apiacas*, *T. obscurus* and *T. silvestris* trigger these manifestations. Therefore, studies on venoms and toxins of scorpions from the Brazilian Amazon are fruitful tools for future research.

### Abbreviations

^1^H NMR: proton nuclear magnetic resonance; 3D: three-dimensional; AC: Acre; AM: Amazonas; AMP: antimicrobial peptide; AP: Amapá; BDTD: Brazilian Digital Library of Theses and Dissertations; BgNa_v_: Na_v_ from the German cockroach *Blattella germanica*; CPP: cell penetrating peptide; EAG: *ether-à-go-go* channel; hERG: the human *Ether-à-go-go*-related gene; IBGE: Brazilian Institute of Geography and Statistics; KTx: potassium channel toxin; K_v_: voltage-gated potassium channels; LCA: Lower Central America; LD_50_: lethal dose required to kill 50%; LILACS: Latin American and Caribbean Center on Health Sciences Informational; MA: Maranhão; MIC: minimum inhibitory concentration; MT: Mato Grosso; NaTx: sodium channel toxin; Na_v_: voltage-gated sodium channels; NDBP: non-disulfide bridged peptide; NF-(B: nuclear factor-kappa B; PA: Pará; PMSF: phenylmethylsulfonyl fluoride; PRISMA: Preferred Reporting Items for Systematic Reviews and Meta-Analyses; Ref.: reference; RO: Rondônia; RR: Roraima; SDS-PAGE: sodium dodecyl sulfate-polyacrylamide gel electrophoresis; SINAN: Brazilian Notifiable Diseases Information System; SIV: simian immunodeficiency virus; TO: Tocantins; VdNa_v_: Na_v_ from the mite *Varroa destructor*; VHL: Virtual Health Library. 
